# A novel high-throughput single-molecule technique DNA curtain: Applications for DNA metabolism

**DOI:** 10.1016/j.mocell.2025.100224

**Published:** 2025-05-20

**Authors:** Soyeong An, Youngseo Kim, Ja Yil Lee

**Affiliations:** 1 Department of Biological Sciences, Ulsan National Institute of Science and Technology, Ulsan 44919, Republic of Korea; 2 Institute of Basic Science Center for Genomic Integrity, Ulsan 44919, Republic of Korea

**Keywords:** Chromatin dynamics, DNA curtains, DNA damage repair, Single-molecule imaging

## Abstract

The advancement of single-molecule imaging techniques has significantly enhanced our understanding of biomolecular reactions and cellular processes that remain obscured in ensemble measurements. In particular, DNA curtains are high-throughput hybrid methods integrating total internal reflection fluorescence microscopy, lipid fluidity, microfluidics, and nano-fabrication, enabling the direct visualization of protein-DNA interactions in real time. The techniques have emerged as powerful tools for probing molecular dynamics of diverse DNA metabolic processes, including DNA damage repair and chromatin dynamics. This review not only highlights recent applications of DNA curtain techniques for elucidating mechanisms underlying DNA damage repair and chromatin dynamics, but also shows how DNA curtain techniques have provided novel insights into the interplay between DNA metabolic processes in the chromatin context.

## INTRODUCTION

Advance of single-molecule imaging techniques has not only revealed molecular mechanisms underlying diverse biomolecular reactions that are hidden in traditional ensemble measurements but also enhanced our understanding of biological phenomena (Kang et al., 2022). Among many single-molecule techniques, the DNA curtain technique has emerged as a powerful tool for elucidating the molecular dynamics of DNA metabolic reactions, in particular DNA damage repair and chromatin dynamics (Cha and Lee, 2021, Kim et al., 2024a). DNA repair plays important roles in genome maintenance and normal inheritance (Jeggo et al., 2016). Defects in DNA repair pathways are highly associated with serious diseases (Jeggo et al., 2016). Furthermore, in eukaryotic cells, DNA is condensed into chromatin, which dynamically changes (Fierz and Poirier, 2019). DNA repair in eukaryotes should be performed in the chromatin context and hence is highly linked to chromatin dynamics. This review surveys the recent applications of DNA curtain techniques for DNA repair and chromatin dynamics.

## DNA CURTAIN TECHNIQUES

**Fig. 1:**
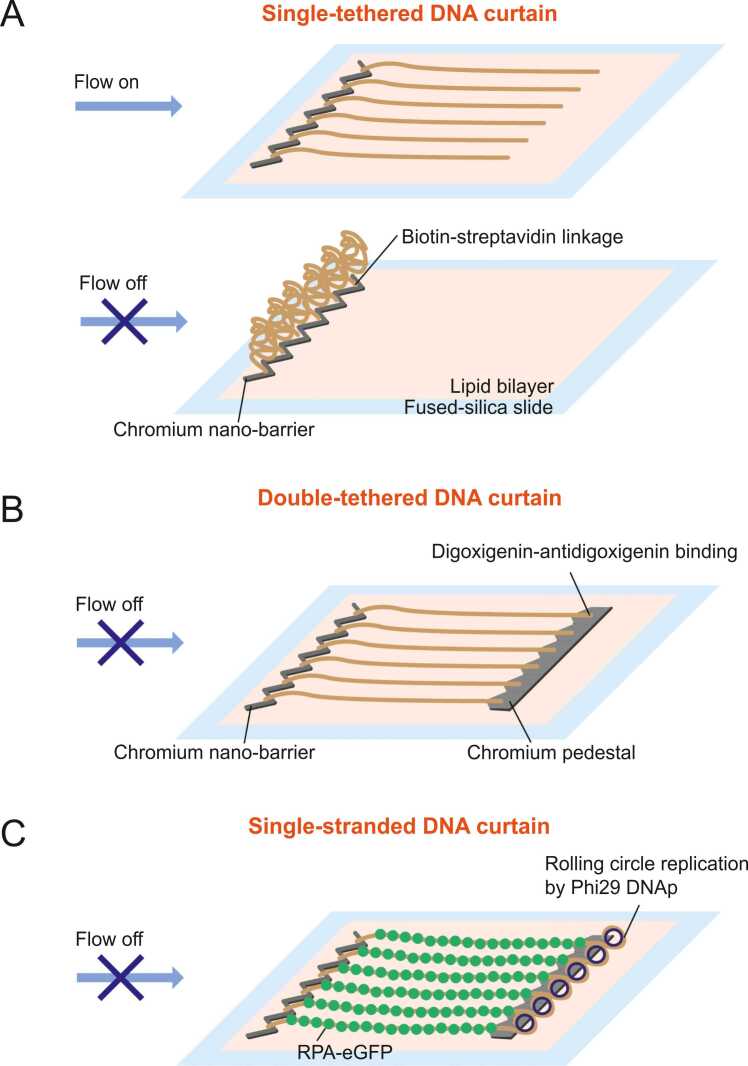
Schematics of DNA curtain assays. (A) Schematic of a single-tethered DNA curtain. A diffusion barrier made of chromium is fabricated on a fused-silica slide, which is covered with a lipid bilayer. DNA molecules are anchored on the lipid via a biotin-streptavidin linkage. Hundreds of DNA molecules become aligned and stretched at the barrier under hydrodynamic flow (top). When the flow is turned off, the DNA molecules recoil due to the lack of a second tether (bottom). (B) Schematic of a double-tethered DNA curtain. One end of DNA, which is anchored on the lipid bilayer via a biotin-streptavidin linkage, is stalled at a diffusion barrier, while the other end is tethered to a pedestal through an antigen-antibody interaction. This configuration ensures that DNA molecules remain stretched even in the absence of flow. (C) Schematic of a single-stranded DNA (ssDNA) curtain. Long ssDNA is generated through rolling circle replication by Phi29 DNA polymerase with a biotinylated primer and then anchored to the lipid bilayer via a biotin-streptavidin linkage. The ssDNA molecules are aligned at the diffusion barrier and subsequently unfolded by RPA (replication protein A), which is tagged with a fluorescent protein and nonspecifically adsorbed to the pedestals. This results in a stable and extended ssDNA curtain even in the absence of flow.

DNA curtain is a high-throughput single-molecule imaging technique combining total internal reflection fluorescence microscopy, lipid fluidity, microfluidics, and nano-fabrication (Cha and Lee, 2021, Kim et al., 2024a). The technique is classified into single-tether and double-tether systems. In the single-tether configuration, only one diffusion barrier, such as a nano-trench or a positive barrier made of chromium or hydrogen silsesquioxane on the slide, is used (Fazio et al., 2012, Kang et al., 2020). DNA is anchored on the lipid bilayer via a biotin-streptavidin linkage. Under buffer flow, DNA molecules on the lipid bilayer align along the flow direction and become stretched at the diffusion barrier (Fig. 1A). When the flow is switched off, DNA molecules recoil out of the evanescent field (Fig. 1A). The hydrodynamic force due to the flow applies only a low force (< ∼4 pN) to DNA. To exert higher tension on DNA, the single-tethered DNA curtain is combined with optical tweezers, enabling single-molecule fluorescence imaging in the presence of tension on DNA (Lee et al., 2012b).

In contrast, the double-tethered DNA curtain system incorporates both a diffusion barrier and a pedestal coated with antibodies (Lee et al., 2012a, Lee et al., 2014). In this configuration, one end of the DNA is modified with biotin, while the other end is labeled with an antigen. Under flow conditions, the biotinylated end is stalled at the diffusion barrier, and the antigen-modified end is tethered to the pedestal, ensuring DNA molecules remain stretched even in the absence of flow (Fig. 1B).

Furthermore, a single-stranded DNA (ssDNA) curtain has been developed to facilitate the study of proteins that interact with ssDNA (Lee et al., 2015, Lee et al., 2016, Lee et al., 2017). Long ssDNA is generated through rolling circle replication using Phi29 DNA polymerase with a biotinylated primer. The ssDNA is anchored on the lipid bilayer via a biotin-streptavidin linkage. Replication protein A (RPA), a eukaryotic ssDNA binding protein, labeled with eGFP (enhanced green fluorescent protein) is introduced to unfold and stretch the ssDNA. The RPA-coated ssDNA is then tethered to the pedestal nonspecifically, allowing the formation of a stable ssDNA curtain even in the absence of flow (Fig. 1C).

### DNA Curtains for Nucleotide Excision Repair

The DNA curtain technique provides a powerful platform for studying DNA damage repair mechanisms. It has been extensively used for investigating the damage recognition mechanism of nucleotide excision repair (NER) (Cheon et al., 2019). Damage recognition is a crucial step for DNA repair process as it serves as the initiation phase and a rate-limiting step. NER mends diverse types of DNA damage, including ultraviolet-induced photo-lesions, bulky chemical adducts and intrastrand crosslinks (Kusakabe et al., 2019, Marteijn et al., 2014). Defects in NER cause xeroderma pigmentosum with serious skin trouble from sunlight and Cockayne syndrome and trichothiodystrophy with mental disorder and delayed developmental (de Boer and Hoeijmakers, 2000, Marteijn et al., 2014). NER operates via two distinct pathways based on damage recognition: global-genome NER, in which XPC (xeroderma pigmentosum group protein C), which forms a complex with RAD23B, detects DNA damage, and transcription-coupled NER, in which stalled RNA polymerase marks a DNA lesion (Schärer, 2013, Sugasawa et al., 1998, van den Heuvel et al., 2021). In global-genome NER, the versatility of XPC-RAD23B lies in its ability to recognize DNA helix distortion caused by DNA damage and binds to the strand opposite to the lesion.

**Fig. 2:**
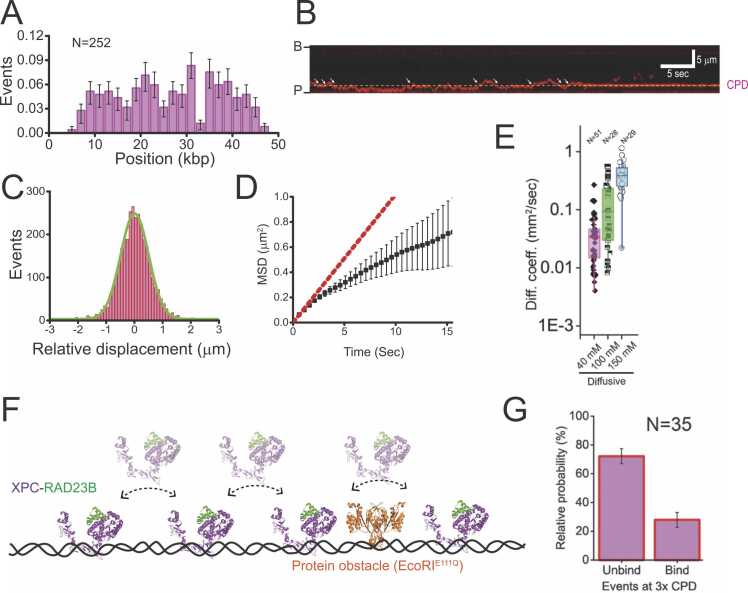
Damage search mechanism of XPC-RAD23B in nucleotide excision repair (NER) using DNA curtains. (A) Histogram for the initial binding positions of XPC-RAD23B on undamaged λ-DNA. Error bars represent the 70% confidence interval obtained through bootstrapping. (B) Representative kymograph illustrating diffusive motion of XPC-RAD23B and its recognition of a DNA lesion (cyclobutane pyrimidine dimer [CPD]). The positions of the diffusion barrier (B) and pedestal (P) are denoted on the left, while the CPD site is marked with a magenta arrow on the right. (C) The histogram of relative displacements between adjacent time frames, extracted from the time trace. The histogram was fitted with a single Gaussian function centered near zero. (D) MSD for the diffusive motion. The diffusion coefficient (*D*_*diff*_) was estimated from the linear fitting (red dotted line) of the first 3 points. (E) Box plots of diffusion coefficients (*D*_*diff*_) according to NaCl concentrations. N is the total number of molecules analyzed. (F) Model depicting the diffusion mechanism of XPC-RAD23B. The protein moves along DNA via hopping rather than sliding, allowing it to bypass protein obstacles efficiently during the damage search process. In experiments investigating XPC-RAD23B collisions, catalytically inactive *Eco*RI^E111Q^ is used as a protein obstacle. (G) The probability that XPC-RAD23B binds to or bypasses CPDs upon encountering the lesions. The binding probability shows the CPD recognition efficiency of XPC-RAD23B during its diffusion at CPD sites. Error bars represent the standard error.

Using DNA curtains, Cheon et al. (2019) demonstrated that XPC-RAD23B initially binds to random sequences on DNA and subsequently undergoes random movement along the DNA (Fig. 2A and B). The distribution of relative displacements of XPC-RAD23B fits well to a single Gaussian function, consistent with one-dimensional (1D) Brownian diffusion (Fig. 2C). Moreover, the mean square displacement (MSD) exhibited a sublinear increase over time, indicative of the constrained one-dimensional diffusion (1D diffusion) (Fig. 2D) because XPC-RAD23B moves on double-tethered lambda DNA. Diffusion coefficients were obtained from linear fits to the initial MSD data points with minimal error (Fig. 2D). Interestingly, the diffusion coefficients of XPC-RAD23B increased with higher ionic strength, suggesting that XPC-RAD23B primarily moves along DNA via hopping rather than sliding (Fig. 2E). Additional experiments revealed that XPC-RAD23B could bypass a protein obstacle, catalytically inactive *Eco*RI^E111Q^, with approximately 30% probability, further supporting the hopping mechanism. These findings provide strong evidence that a hopping protein can search for its target by bypassing proteins bound to DNA (Fig. 2F). Finally, the study demonstrated that XPC-RAD23B identifies a photo-lesion, cyclobutane pyrimidine dimers (CPD), through the diffusion. However, the CPD recognition efficiency of XPC-RAD23B was relatively low (∼8.6%) due to little DNA distortion by CPD (Fitch et al., 2003, Sugasawa et al., 2001) (Fig. 2B and G). Therefore, for sensing CPDs, another protein, UV-DDB (ultraviolet damaged DNA-binding protein), is necessary.

(A-D, F) This figure is adopted from Nucleic Acids Research (Cheon et al., 2019). (e) The structures of XPC-RAD23B (PDB ID: 2QSH) and *Eco*RI (PDB ID: 1ERI) are adopted from the Protein Data Bank.

### DNA Curtains for R-loop Search Mechanisms of Tonicity-responsive Enhancer Binding Protein

**Fig. 3:**
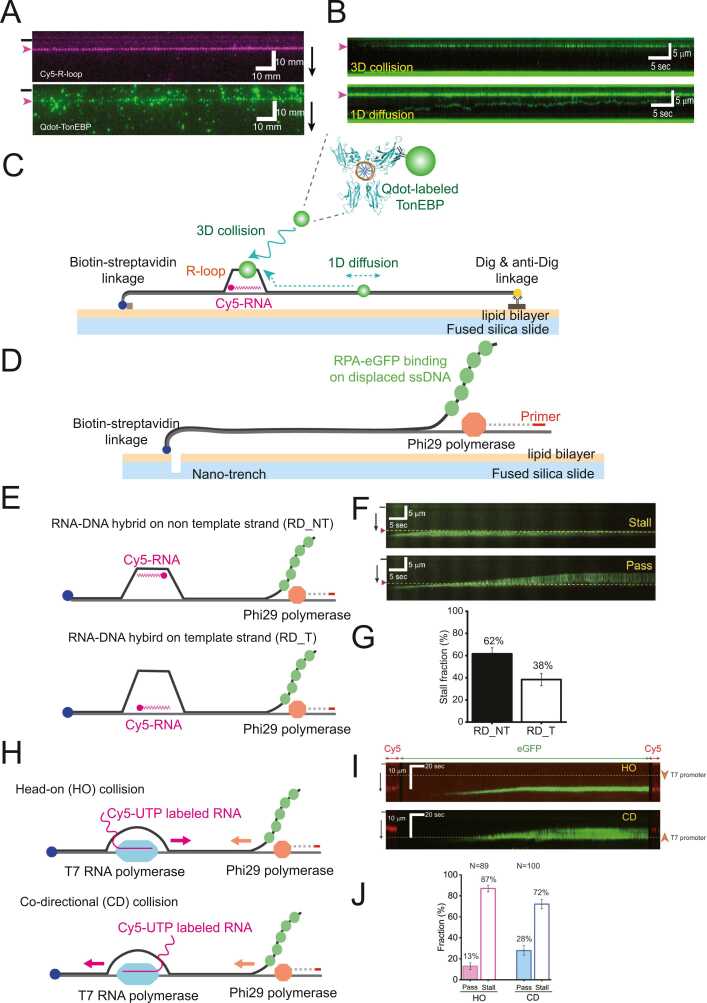
DNA curtains for R-loops. (A) Single-tethered DNA curtain images showing (top) an R-loop consisting of Cy5-labeled RNA and (bottom) TonEBP labeled with quantum dot (Qdot). Black bars and magenta arrows on the left indicate the positions of the diffusion barrier and the R-loop, respectively. Black arrows on the right denote the direction of buffer flow. (B) Representative kymographs illustrating the R-loop search mechanisms of TonEBP via (top) three-dimensional (3D) collision and (bottom) 1D diffusion. Magenta arrows on the left mark the R-loop positions. (C) Schematic illustrating the R-loop search mechanisms of TonEBP using DNA curtains. Qdot-labeled TonEBP identifies R-loops along DNA through two distinct mechanisms: 1D diffusion and 3D collision. In addition, TonEBP preferentially binds to the displaced ssDNA of R-loops. (D) Schematic of DNA curtains for visualizing Phi29 DNA polymerase replication. In the single-tethered DNA curtain system featuring nano-trenches, Phi29 polymerase initiates replication from a primer annealed to the free DNA end. As DNA synthesis progresses, Phi29 DNA polymerase unwinds the duplex DNA, and the displaced ssDNA is subsequently coated with RPA-eGFP. The replication process is monitored in real time by tracking RPA-eGFP fluorescence. (E) Schematic of collisions between an R-loop and Phi29 DNA polymerase, depending on the position of the RNA-DNA hybrid within the R-loop. RNA is annealed to (top) the nontemplate stand (RD_NT) or (bottom) the template stand (RD_T). (F) Representative kymographs showing the behavior of Phi29 DNA polymerase upon encountering an R-loop. The polymerase either stalls or bypasses the R-loop. (G) Stall probability of Phi29 DNA polymerase at R-loops, which varies with the strand involved in RNA-DNA hybrid formation. When the RD is located on the nontemplate strand (RD_NT), the polymerase stalls in ∼62% of events, whereas stalling occurs in ∼38% of events when the RD is on the template strand (RD_T). (H) Schematic of collisions between Phi29 DNA polymerase and T7 RNA polymerase in either (top) a head-on or (bottom) codirectional orientation. (I) Representative kymographs displaying (top) the behavior of Phi29 DNA polymerase upon encountering T7 RNA polymerase. The polymerase stalls (top) in the head-on orientation, whereas it bypasses (bottom) in the codirectional orientation. (J) Quantification of pass and stall probabilities for Phi29 DNA polymerase upon collision with T7 RNA polymerase.

R-loops are 3-stranded nucleic acid structures, consisting of an RNA-DNA hybrid (RD) and a displaced ssDNA (Cheon et al., 2022). They play important roles in various nuclear processes and cellular activities, such as gene expression, chromosome segregation, immunoglobulin class switching, telomere regulation, and immune activation (Cheon et al., 2022). Despite their essential functions, excessive accumulation of R-loops can lead to genomic instability (Li et al., 2023, Uruci et al., 2021). R-loops interfere with replication fork progression, causing replication stresses, while the exposed ssDNA in an R-loop serves as a target for endonucleases. Unregulated R-loops are associated with diseases such as cancer and neurological disorders (Cheon et al., 2022, Hwang et al., 2024). Recent studies have shown that R-loop resolution is regulated by the tonicity-responsive enhancer binding protein (TonEBP) (Cheon and Lee, 2022, Kang et al., 2021a). TonEBP, known as NFAT5 (nuclear factor of activated T cells 5), is originally a transcription factor that regulates the expression of inflammatory genes in immune cells, such as macrophages and T cells. TonEBP is also included in the expression of osmo-protected genes under hypertonic stress (Choi et al., 2020, Roth et al., 2010). In the study, DNA curtains visualized preferential binding of TonEBP to an R-loop (Fig. 3A). Furthermore, it was revealed that TonEBP finds an R-loop through both 1D diffusion and three-dimensional (3D) collision, suggesting that this dual-mode search mechanism facilitates efficient scanning of the long human genome for R-loop search (Fig. 3B and C). Additional biochemical assays demonstrated that TonEBP preferentially binds to the displaced ssDNA, further supporting its role in R-loop recognition and resolution (Cheon et al., 2022, Cheon and Lee, 2022, Kang et al., 2021a). Based on laser microirradiation assays, TonEBP interacts with the METTL3-METTL14 complex, a methyltransferase responsible for catalyzing N^6^-methyladenosine (m^6^A) RNA modifications. These findings suggest a novel mechanism for R-loop resolution, by which TonEBP, bound to the displaced ssDNA of R-loop, recruits the METTL3-METTL14 (methyltransferase-like 3 and methyltransferase-like 14) complex. The subsequent deposition of m⁶A on the RNA strand facilitates R-loop resolution through RNaseH1-mediated RNA degradation (Kang et al., 2021a).

(A and B) The figures are adopted from Nucleic Acids Research (Kang et al., 2021a). (F, G, I, and J) The figures are adopted from Nucleic Acids Research (Kim et al., 2024b). (c) The TonEBP structure (PDB ID: 1IMH) is adopted from the Protein Data Bank.

### DNA Curtains for the Collision Between Replication and R-loop

R-loops have long been suspected to obstruct replication fork progression, but direct evidence for replication stalling by R-loops has remained elusive. Kim et al. directly visualized the collision between replication machinery and an R-loop using a single-tethered DNA curtain system (Kim et al., 2024b). In these experiments, biotinylated lambda DNA containing a Cy5-labeled R-loop inside was immobilized on lipid bilayer, while the other free end was primed for replication initiation by Phi29 DNA polymerase. Upon the addition of dNTPs, the polymerase began to unwind the duplex DNA and to synthesize new DNA along a single strand. The replication progression of Phi29 DNA polymerase was monitored using RPA-eGFP bound to the displaced ssDNA (Fig. 3D). When Phi29 DNA polymerase encountered an R-loop, it either stalled or bypassed the R-loop, showing that a single R-loop can indeed impede replication in vitro (Fig. 3F). Interestingly, the stalling probability varied depending on which strand the RD formed. When the RD was on the nontemplate strand, the stalling fraction was ∼62%, which was higher than that (∼38%) for the RD on the template strand (Fig. 3E and G). The authors proposed that secondary structures on the nontemplate strand interfere with the bending of the displaced ssDNA, inhibiting Phi29 DNA polymerase progression. They further studied the collision between transcription of T7 RNA polymerase and replication by Phi29 DNA polymerase (Fig. 3H), showing that head-on collisions caused more significant replication blockage than codirectional collisions (Fig. 3I and J). Collectively, these findings underscore the critical role of R-loops in replication stress and provide novel insights into the mechanisms by which R-loops interact with replication machinery.

### DNA Curtains for Chromatin Dynamics

**Fig. 4:**
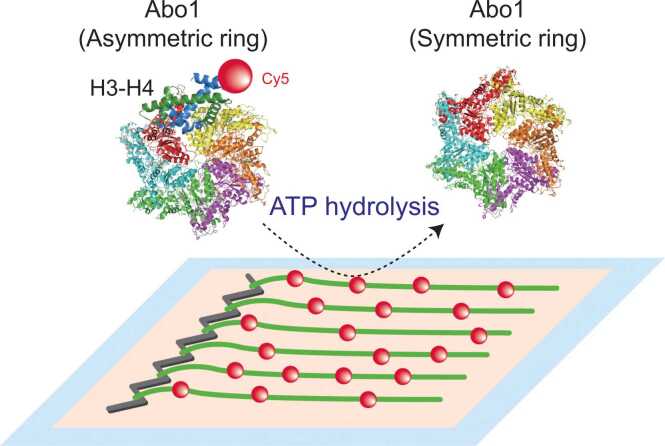
Function of *S. pombe* AAA+ ATPase Abo1 revealed by DNA curtains. Abo1, a bromodomain-containing AAA+ ATPase in *S. pombe*, assembles into a homo-hexamer and undergoes ATP-dependent conformational changes. When ATP is bound, Abo1 adopts an asymmetric spiral conformation (left, PDB ID: 6JQ0), whereas it transitions to a symmetric ring structure (right, PDB ID: 6JPU) in non-ATP state. DNA curtain studies reveal that Abo1 facilitates the ATP-dependent loading of histone H3-H4 dimers (PDB ID: 7Y5W) onto DNA along with its conformational change.

Nucleosome assembly and disassembly are catalyzed by histone chaperones, such as Asf1 (anti-silencing factor 1), CAF-1 (chromatin assembly factor-1), and FACT (facilitates chromatin transcription) (Hammond et al., 2017). These histone chaperones facilitate histone dynamics without utilizing chemical energy from ATP hydrolysis. However, some ATPases, including bromodomain-containing AAA+ ATPases, have been reported to function as histone chaperones. Among them, ATAD2 (ATPase family AAA domain-containing protein 2) is known to mediate the loading and removal of histone H3-H4 and is frequently overexpressed in various cancers, where it is associated with poor prognosis (Caron et al., 2 010, Kalashnikova et al., 2010, Zou et al., 2007). Unlike ATAD2, genetic studies for its *Schizosaccharomyces pombe* homolog, Abo1, have shown that Abo1 promotes nucleosomal density by facilitating H3-H4 loading (Gal et al., 2016). Cho et al. revealed that Abo1 forms a homo-hexameric ring structure and ATP binding induces a conformational change from a symmetric to an asymmetric ring (Cho et al., 2019b). DNA curtain assays further revealed that Abo1 does not disassemble tetrasomes or nucleosomes but loads H3-H4 onto DNA in an ATP-dependent manner (Fig. 4) (Cho et al., 2019a). This loading process is sequence-independent and involves transient interaction between Abo1 and DNA (Kang et al., 2021b).

Moreover, Abo1 does not incorporate H2A-H2B dimers, which are instead deposited onto tetrasomes by FACT (Formosa and Winston, 2020). The interaction between Abo1 and FACT was also investigated using single-molecule imaging (Jang et al., 2025). DNA curtains revealed that FACT does not modulate Abo1′s histone loading activity nor cooperates in nucleosome assembly with Abo1. Instead, single-molecule particle counting showed that Abo1 facilitates the dissociation of FACT from nucleosomes in an ATP-dependent manner without disrupting tetrasomes or H2A-H2B dimers. These findings were corroborated by biochemical assays, such as electrophoretic mobility shift assay, a method valued for its simplicity, speed, and capability to test multiple conditions simultaneously (Jang et al., 2025). Consistent with the single-molecule imaging data, electrophoretic mobility shift assay demonstrated that Abo1 dissociates FACT from the nucleosomes, which were reconstituted with tetrasomes and H2A-H2B dimers together with FACT, while it leaves the nucleosomes intact. Importantly, this activity was ATP-dependent, as Abo1 failed to remove FACT in the absence of ATP. This new function of Abo1 that dissociates FACT from nucleosomes facilitates to recycle FACT.

This new function of Abo1 that dissociates FACT from nucleosomes facilitates to recycle FACT.

## TECHNICAL LIMITATIONS OF DNA CURTAIN ASSAY

Although DNA curtain techniques have been widely utilized to investigate diverse DNA metabolic reactions as reviewed above, they present several technical limitations. While DNA curtains are particularly powerful for visualizing long-range protein movement, their spatial resolution (∼1 kbp per pixel) precluded the detection of protein dynamics within shorter DNA segments. For studying conformational changes or motions on the nanometer scales, single-molecule FRET (fluorescence resonance energy transfer) technique offers a complementary approach. However, there remains a need for new techniques capable of probing biomolecular interactions in the intermediate range of several to hundreds of nanometers. Another limitation of DNA curtains arises from the configuration of the assay. DNA molecules are stretched close to the surface. Although lipid bilayer passivation reduces nonspecific interactions, surface effects may still influence protein-DNA interactions. To circumvent these effects, DNA tightrope and DNA sky-bridge platform have been proposed as an alternative that elevates DNA away from the surface (Jang et al., 2019, Kim et al., 2019).

While curtain techniques have primarily been developed for DNA, future advancements in RNA curtains will be essential for studying interactions between RNA and RNA binding proteins as well as the dynamics of ribosomal translation.

## CONCLUSION

The DNA curtain technique has established itself as a strong tool in single-molecule imaging, enabling high-throughput visualization of protein-DNA interactions. This review has highlighted its significant contributions to our understanding of DNA repair, chromatin dynamics, and genome maintenance. As technological advancements continue to refine and expand the capabilities of DNA curtains, their integration with complementary techniques, including super-resolution microscopy and single-molecule force spectroscopy, will further illuminate the complex interplay of proteins and nucleic acids in living cells. The continued development and application of DNA curtain techniques will undoubtedly drive new discoveries in DNA metabolism, offering potential therapeutic implications for diseases linked to genomic instability.

## Author Contributions

**Soyeong An:** Writing – original draft. **Youngseo Kim:** Writing – original draft. **Ja Yil Lee:** Writing – review & editing, Writing – original draft.

## Declaration of Competing Interests

The authors declare no conflicts of interest.
